# The New York Institute of Stomatology

**Published:** 1902-11

**Authors:** 

**Affiliations:** The New York Institute of Stomatology


### Reports of Society Meetings.

### THE NEW YORK INSTITUTE OF STOMATOLOGY.

A MEETING of the Institute was held at the office of Dr. G. S. Allan, No. 51 West Thirty-seventh Street, New York, on Tuesday evening, May 6, 1902, the President, Dr. J. Morgan Howe, in the chair.

The minutes of the previous meeting were read and approved.

*The President.*—The following report has been received from the committee appointed to prepare a memorial to be entered on the minutes regarding the death of our first president, Dr. Benjamin Lord.

"The New York Institute of Stomatology, having heard with deep regret of the death of our honored friend and first president, Dr. Benjamin Lord, desires to express in this formal way our sense of loss and bereavement.

"Dr. Lord, by his earnest and painstaking devotion to the pursuit of the ideal of excellence, had won the respect and esteem of all who knew him.

"He was one of the organizers of this Institute, and its earnest friend to the last. The raising of a fund for scientific investigation in our branch of the healing art, which fund has been placed in the hands of the Board of Directors of the Institute, to be used at their discretion, was mainly due to his efforts.

"His more personal qualities, his firmness of opinion, his loyalty to the truth as he perceived it, his frank outspokenness, no less than his kindly feeling, have endeared him to his friends and made him a force in their counsels.

"Our profession has been honored by so sound and true a life, the key-note of which has been the faithful doing of daily duty, and we are all the poorer by the loss of such a friend, who, having 'been faithful in a few things,' has heard the 'well done' of the Master and has entered into the joy of the Lord."

A paper written by Dr. E. Lloyd-Williams, of London, was read by Dr. Kimball.

(For Dr. Lloyd-Williams's paper, see page 794.)

The secretary read a letter from Dr. Barnes, of Cleveland, Ohio, expressing his interest in the subject of the evening, and the belief that dentists would prove of great value on hospital staffs.

*The President.*—I have now the pleasure of introducing our fellow-member, Dr. Flanagan, of Springfield, Mass., who will read a paper entitled "The Hospital's Need of a Dental Staff."

(For Dr. Flanagan's paper, see page 796.)

DISCUSSION.

*Dr. R. C. Newton.*—Mr. President and gentlemen, if I should say that I feel greatly honored at the invitation to be present and take part in the discussion here to-night, my words would but feebly express my feelings. I am much pleased to be here and much pleased to have an opportunity to help in any way that I can a movement in which for some years I have taken an especial interest.

I agree fully with the contention of Dr. Flanagan's paper that hospital work would be better and more complete if dental surgeons were represented on all hospital boards. There is no question that such a state of affairs would benefit the patients in the hospitals. To my mind it is equally clear that it would benefit the medical men connected with the hospitals, both the internes and the externes; and lastly, it could not fail to benefit the dentists themselves. On the latter aspect of the question, at the risk of seeming to be personal, I will make, if you will permit me, a few observations. Perhaps I cannot do better in stating my position than to quote from an address delivered before the Section on Oral and Dental Surgery of the American Medical Association in 1895, by Dr. Latham, of Chicago. The doctor says, "Every member of the dental profession can recall the days of his service in the operating-room, and remember his difficulty in procuring help from the professor or his assistants. For, as a rule, the corps of instructors in our colleges, medical and dental, is much too small." I might say, in passing, that when I was a medical student these strictures would hold true against the medical school where I studied my profession. It was an unendowed, inadequately manned, and poorly managed institution, housed in a dingy, ill-ventilated, and cramped building, where a proper oversight of the students was not exercised, and where the personal help and guidance which every student needs had to be obtained from private quiz-masters. Such was the best medical school in the city of New York twenty-five years ago; and now see what it has become, as the Medical Department of Columbia University. The great and crying need for better quarters, more teachers, better laboratory facilities, has been heard, and you all know the present fine equipment not only of that medical school, but of all the principal medical schools in this city and elsewhere. I mention these facts to encourage you. Your dental schools must be improved and enlarged. They must be endowed and be called part of the great universities. If you say that this will be impossible, I point you to what has been done for the medical schools. It is not impossible. It is not only possible, but, for one, I fully believe that it will come. However, I anticipate. I will, with your kind permission, return to our author's address, which continues:

"The larger the clinics, the more benefit we may derive from them. The larger the corps of capable demonstrators, the more practical and successful professional men and women we become, It seems a curious fact that works on dental surgery are so imperfect and rambling on the subject of diagnosis."

Why are dentists, as a class, not better diagnosticians? Dr. Latham gives five reasons as follows:

"(1) The hurry to obtain a diploma.

"(2) The study of only just what seems absolutely necessary in the practice of dentistry, and a corresponding inability to apply general principles.

"(3) The fear of encroaching on general medicine.

"(4) Insufficient preliminary education.

"(5) Lack of a thorough knowledge of the normal conditions and a habit of relying too much upon one mode of treatment.

"A student may be well versed in the sciences of his profession, and yet unable to diagnose the simplest case of disease presented in actual practice."

Admitting that the above remarks are true, it seems doubtful whether the average dentist will be satisfied to continue in this inferior position. Will he not rather take every means at his command to improve the morale and the equipment of his profession, until no one shall say that intellectually or socially he stands below the average practitioner of medicine? If, as I said above, dentists shall be appointed on the staffs of general hospitals, numerous advantages would follow.

The first, and perhaps the most important, influence would be upon the internes. I have long believed that my profession is somewhat lax in studying sufficiently and giving due consideration to the condition of the teeth of their patients. Our text-books have little to say upon this important subject. Too much is left to the discretion of young practitioners, and, as we have often observed, the condition of the teeth may be overlooked or ignored. All well-equipped medical men will admit, if they are asked, that unless the denture be good and the patient takes the time and trouble to masticate his food properly, dyspepsia, constipation, neurasthenia, with all their never-ending train of symptoms, leading in many cases to complete destruction of the physical and mental health, will surely follow.

On all of our book-shelves are heavy volumes on diet; we spend much time in determining the relative amounts of proteids, hydrocarbons, and carbohydrates which the ideal dietary calls for; but there is very little time or trouble spent in instructing people how to eat and how to care for their teeth. The value of teeth as such is not sufficiently impressed upon the minds of the common people. Very fortunately fashion leads many, especially women, to be exceedingly careful of their teeth and to spare no pains or expense in their preservation. However, the younger doctors themselves seem to me to be far too careless not only about their own teeth, but in instructing their patients to care for theirs. In administering some of the almost interminable list of remedies for dyspepsia, how often do we think to carefully explore the patient's mouth and ascertain whether he has the facilities for properly masticating his food and mixing it with saliva before it goes into the stomach at all? I have seen eminent medical men bolt their food practically unchewed, so that it enters the stomach in an unfit condition and can never be properly digested and assimilated. If these men have ever had the necessity of eating properly impressed upon them, they have got far away from their teaching.

I believe, for this reason alone, it would be very desirable if young medical men and young dentists mingled together during their student days, that the value of the teeth and how to preserve them might be impressed upon the physician more strongly than it seems to be at present.

As to the benefits which would accrue to the patients in hospitals if there were some dentists around to take care of their teeth, I need not take up your time by arguing the question; we all agree that the benefit to the patients would be incalculable. Therefore let us turn our attention for a few moments to some of the advantages which young surgeons might reap from a hospital course. I have already touched upon some of the present disadvantages under which dental education suffers. Like all specialists, the field of dental work being somewhat limited, dentists tend to grow narrow and one sided, to think only of the teeth of their patients, to forget that there are other organs in the body and that a comprehensive view of medicine and hygiene must include a wise care of them all.

There seem to be some superstitions surrounding dental practice, just as there are ordinary every-day medicine. Perhaps you will forgive me if I mention a little incident which came to my knowledge a day or two ago. A young physician was sent for to lance the teeth of a squalling infant. He read the mother a lecture on interfering with the ways of Divine Providence, informing her that God never meant teeth to be lanced, and then directed her to rub the teeth through with her thimble. The incongruity of his utterances never seemed to strike him. But one would think that in our day an aseptic, well-sharpened lance would be preferred to a rough, dirty thimble. However, this young gentleman had not enjoyed a liberal preliminary education. From lack of the mental balance and acumen which comes from study and experience, the absurdity of his advice did not appear to him; and so with many a doctor who lets prejudice or pique take the place of reason and judgment. This very matter of lancing gums in teething infants meets frequently with this objection from doctors, that if the cut heals over it will be harder for the tooth to come through, because scar tissue is more resistant than normal tissue. This reason is at variance with the facts, which are just the reverse, as a moment's reflection will show any one who is at all familiar with pathology. Whatever view a practitioner may take of the procedure, the objection commonly alleged against it is not well founded, and shows the unthinking way in which many physicians decide medical questions.

In the same way dentists seem to cherish some ancient superstitions. I will mention two or three of those that I have most commonly met with.

Dentists not seldom decline to draw teeth in the height of a toothache from greatly swollen gums, giving the patient to understand that it will be dangerous or impracticable to do so, thus adding, providing of course that the tooth must be sacrificed eventually, several hours' or days' pain and discomfort needlessly to the patient's suffering. Again, certain dentists decline to fill or clean the teeth of pregnant women, thinking that this will bring on a miscarriage. While it is perfectly conceivable that dental work might bring on a miscarriage, I believe that it has very rarely, indeed, done so, and in the vast majority of cases it is far better to preserve the teeth of a pregnant woman by doing the necessary work to them than to allow the caries to go on until after the confinement is over, and so not only subject the woman to the risk of losing the teeth altogether, but also adding to the gastronomic discomfort and indigestion from which a pregnant woman generally suffers by leaving decayed and probably painful teeth in her mouth. My experience leads me to assert that the shock of conception and the early changes in the economy following it are especially marked in their destructive influence upon the teeth. In other words, shortly after a woman discovers that she is pregnant she begins to complain of her teeth, new cavities form and old ones enlarge, so that the fillings not infrequently drop out. The old dictum that odontalgia is a sign of pregnancy is too generally accepted to be without foundation. Now, to decline to interfere with this destructive process for several months is, in my opinion, poor surgery and worse dentistry.

Again, the present day may be said to be the era of dieting. The medical profession is forbidding this and avoiding that, until it seems difficult to find any form of alimentation that is at once nourishing and innocuous; and now come the dentists and forbid this and that article of diet, especially at present, I believe, grapefruit, as its acid properties are asserted to be destructive to the teeth. And inasmuch as uric acid in the body is alleged to be one of the causes, if not the principal cause, of Rigg's disease, of recession of the gums, etc., and under the assumption that vegetable acids are formed into, or augment the formation of, uric acid, they must be forbidden; because they may cause rheumatism and subsequently gingivitis, pyorrhœa, alveolaris, etc. This I esteem

"bad medicine" and unwonted interference with the natural and proper craving of all human beings, and especially the young female, for acids. If bad effects can be shown to follow the use of acids in any particular case (and there are people whose systems will not tolerate acids), let them be forbidden for that case. But to forbid all people to eat grape-fruit because its acid may have injured the teeth of a few, is in my opinion unwarranted.

The question of diet is an exceedingly complicated one. The best men in medicine are by no means agreed upon the rules which should govern it, nor upon the ideal dietary. What is sought to be accomplished by withholding ailments of one class may be gained at a great injury to the economy generally, and for this reason a too rigid diet is often quite hurtful, and may do more harm than good. Therefore the dentist must be careful and conservative in undertaking to enforce rules about which experts are still in doubt. These are only a few of the questions, Mr. President and gentlemen, which must come before the conscientious dentist, and which are still puzzling his medical brother. They call for the highest medical education, as well as a great deal of experience and sound judgment. The dental practitioner must himself be able to judge of the merits of each case as it comes before him, and must not be too much influenced by the *ipse dixit* of any teacher, no matter how eminent.

Lastly, if I have not already taken up too much of your valuable time, there is another view of this question which you cannot neglect. I refer to its charitable aspect. Unless dentists are ready and willing to give a certain portion of their time and skill to the poor, dentistry can scarcely be esteemed a part of the liberal profession of medicine. It is the liberality of physicians, the willingness to give up their best skill and thought without any immediate and tangible reward, that has made our profession the most honored and beloved of the learned professions. It is this that gives us an almost unbounded influence. The rich will endow hospitals freely so long as there is need for them; so they would endow dental hospitals and clinics just as soon as there shall be found enough dentists willing to give the time and skill necessary to care for the teeth of the poor. No man can live rightly in our day without trying to help his brother. No man can himself become a broad-minded, philanthropic man, who, seeing his brother have need, will not give him what he alone can give.

Mr. Morgan or Mr. Carnegie may endow many hospitals, but

I doubt whether they can fill teeth properly. It is up to you to supply one of the greatest needs of our modern civilization in caring for the poor man's teeth.

It is no flight of the imagination, but a truth to which I believe every man in this room will assent, that bad teeth (and my experience leads me to say that practically all the very poor have bad teeth), lead directly to intemperance in drink. Improperly masticated food produces chronic dyspepsia, which produces pain, uneasiness, and flatulence in the stomach and bowels, hence the craving for the alleviating effect of alcohol, for the comfort and warmth which follows its ingestion; hence our poor man, not himself recognizing why he craves stimulation, will seek it and get it. He will forget his troubles after a few drinks, and no amount of literature and no number of lectures on the evils of the drink habit will still that gnawing in his stomach, nor prevent him from taking comfort in the only way that he knows when the pains of dyspepsia seize him. Because dental caries is so insidious in its onset and so exceedingly common, the general public and philanthropic people in particular, do not seem to appreciate its extent or the evils to which it gives rise.

This is your day and your opportunity. You must come forward and instruct the laity, and especially those who are laboring for the poor and the criminal classes, on the necessity of doing something for the teeth of these unfortunates, and you yourselves must be willing to supply the skill and labor needed. After many, many years of neglect our government and the English government also are just appointing dentists to look after the teeth of their soldiers. Dentists ought to be appointed not only to the army and navy, but to all jails, asylums, and especially reformatory institutions, where the young and partly grown are detained. To neglect the teeth of the inmates of such institutions is a crying evil whose magnitude is but dimly seen by the wisest, and apparently not thought of by the average philanthropist, not to mention the politician.

Valuable preliminary steps have been taken, mainly, I believe, by the dentists, but only the fringe of the work has been touched. Let us join hands. Let us make the movement which is already shaping itself so well forcible and general. I know how the best men among you feel, and I am sure that whatever you find in your hearts to do for the good of humanity and for the advancement of the healing art, you will do; and you shall have the hearty support and sympathy of your medical colleagues and of thinking men and women everywhere.

You will show the world at large that dentistry is not purely a mechanical art, but a learned profession, and that dentists are both willing and able to do their share to help the downcast and the unfortunate in the way in which only dentists can help them.

> "In Faith and Hope the world will disagree,
> But all mankind's concern is Charity."

*Dr. R. H. M. Dawbarn.*—First of all, let me apologize for having arrived so late, which was not at all my intention. I was delayed at the last moment, as we sometimes have to be, and I am particularly sorry to have missed the papers, save the last. The last paper was by a gentleman whom I have met often and whose eloquence I greatly appreciate. It was a paper that struck home to the very root of this subject. Before I say anything about the hospitals, just a word about this subject of an acid dietary. It is a fact not altogether thoroughly recognized that a diet including vegetable acids is not necessarily followed by an acid saliva. On the contrary, a diet containing vinegar or lemon-juice will cause an alkalinity of the urine, and if it will do so with one secretion I am inclined to think it will do so with the saliva. I do not think we all recognize the value of vinegar as an article of food. It is an historical fact that the Romans, at the time of their supremacy, furnished a daily ration of vinegar to their soldiers; which is the reason why the soldier at the cross was able to give vinegar to the dying Saviour.

As to the hospital question, I do not know that I need to tell you what my sympathies are. The work that I have done in behalf of having dental surgeons appointed to our largest hospital in this city speaks for itself; and what I can say will simply be reiterating what I have already said. The general crux of this whole question is this, Are we to consider ourselves (I use the "we" here partly in the editorial sense and partly because I am an honorary member of this body),—are we to consider ourselves as part of the medical profession in the same sense as the rhinologist, the laryngologist, or the ophthalmologist, or are we to consider ourselves as a distinctly separate profession? It does seem to me that it is in the interest of all parties concerned that the dentists should merge their individuality into that of the medical profession, and to consider themselves as one specialty of that body. I am very sure that this is the destiny of the profession, and that those who oppose it will gradually be swept into the small minority. There is no question but that a man may be an excellent dentist and still not be a physician; but I think it is for the best interests of dentists to be physicians first and dentists afterwards. Every dentist should command two degrees, first the M.D., and second the degree in dentistry.

In my work at the City Hospital I have again and again been struck with the deplorable condition of the teeth of the city's poor. Most of them have decayed teeth and many have ulcerated roots. During the last administration I undertook to induce the Commissioner of Charities to permit me to order tooth-brushes whenever I wished to prescribe them. I tried to convince him that the patients would recover much more quickly from their various medical or surgical ailments because of improved digestion if this were done, and that therefore the city would be in the end the gainer. But I could not induce him to see it in that light. He said that there were several objections, not the least of which was that the patients would in the majority of cases rise up in rebellion; that if they, by improved mouth hygiene, increased their appetites, there was still more trouble, because he was then having all the work he could do to induce the Board of Estimate and Apportionment to scrape together enough food to keep body and soul together. It seemed something after the plan of Mrs. Squeers, who gave the boys brimstone and treacle to keep down their appetites. But this is looking at it from a jocular stand-point. These poor fellows, it seems to me, deserve the services of better men than physicians or surgeons unequipped with your special knowledge. There is not a medical man upon the whole board of this largest of all our hospitals who is able to treat properly such troubles of the mouth as have their origin in the teeth. I determined to reverse this condition of things, and succeeded in inducing the board to appoint two dental gentlemen to the board. Then came the difficult part of what was to follow, and that was to give them equal rank with ourselves. I am certain that the only reason why I succeeded was because both these gentlemen, Dr. Deane and Dr. Bogue, are physicians. I would have been in the hopeless minority if I had endeavored to have men other than M.D.s appointed, however able they might be as dentists. It is the only hospital in Manhattan in which a dentist has a seat and a vote in the board. Even the pathologist of the various hospitals, always an able physician, is not given a vote. But in the City Hospital the dentists now each have a vote.

Now, gentlemen, the question arises whether these dentists are honoring themselves more in accepting the service and its duties or punishing themselves more; but there are two sides to this question. They are giving up a great deal of valuable time, which means money. Dentists have never done that heretofore in the hospitals, but it seems to me, as specialists in medicine, they should recognize their responsibility to the poor, that noblest attribute of our profession, in that we are so willing to give our time to charitable work. I am on duty five months in the year and I give three entire afternoons a week without any recompense whatever; but this is no more than every visiting surgeon does; for there is not a hospital in America, so far as I know, where the visiting physicians or surgeons receive one penny. The dental members are to be on duty all the year, one day a week, which is roughly in the same proportion. They can doubtless, if desired, have assistants appointed to cover the vacation period. I wish to congratulate this society in finding among its members a gentleman who was willing to step forward and give his valuable time. The First District Dental Society is equally to be congratulated. I hope these gentlemen will continue to maintain their positions, even at the great personal sacrifice required.

*Dr. H. W. Gillett.*—I have much appreciated the three papers we have heard to-night. It has been my privilege to put into the hands of one or two gentlemen connected with hospitals a paper on this subject presented some years ago by Dr. Newton, and I think it has served a very good purpose. The problem is a very large one. When it is possible, and we know it to be common enough, for people of wealth and refinement in the city of New York to come into our offices with mouths in a condition which necessarily means disease of the whole system, when these people in the hands of the very best physicians in the city as their private patients can still show this condition, it must be a fact beyond controversy that the diseased conditions of the poor of our hospitals are influenced by the filthy conditions of their entirely neglected mouths. There should be an awakening on the part of the medical as well as the dental profession to the need of our working together. Dr. Newton is one of the comparatively few physicians who have given the conditions of the mouth careful attention. The extreme septic conditions frequently found in the mouths of surgeons and trained nurses is worthy of attention.

I think nothing has been said about the necessary limitations of the work that dentists can do in the mouths of hospital patients. There is more work needed in the mouths of the poor of any city than all its dentists can find time for if they do nothing else. Where are we to begin, and where draw the line? Shall we be satisfied with simply producing a cleanly condition of the mouth, the mere cleansing, and the relief of pain? If so, we must destroy a great many possibilities of usefulness in these mouths. The point has often been made that many of these people cannot even command the time that is necessary for these operations. This will perhaps raise another question,—Can we do more for the younger people? Perhaps we can simply clean up the mouths of adults and give more attention to the younger generation, with an age limit of sixteen or eighteen years. Can we not do some preventive work for these children, with the hope that later they will be able to get into the hands of a private practitioner.

The question of time is one that the medical profession find it difficult to understand. An hour barely serves to begin a simple case for the dentist, while a physician may often prescribe for ten or twenty in the same time. I do not think the dental profession unwilling to undertake charitable work. I think the question before us is to find the opportunity, and I have faith that our profession will do its whole duty.

*Dr. W. St. George Elliot.*—The subject, of course, is a very broad one, and one that I have always had a great deal of interest in. Dentists, as we know them, are good men. They are just as willing as the medical profession to give their time for the good of mankind. The medical profession does not understand the position in which we are placed. It is a point in this matter to learn what our duty is, for when we know our duty we can do it. I applied to St. Mary's Hospital to see if I could be of any service. The attending surgeon was not particularly encouraging, but he had no serious objections. They raised three or four hundred dollars, and the outfit was secured. I began work, and for two years kept it up. The amount of work to be done was enormous. Ninety-five per cent. of those children had decayed teeth. My idea was to treat the children in the same way that I would treat my own patients. I would fill cavities and clean where necessary. The English surgeons have confined themselves largely to extraction, and that is probably as far as we can go. Probably ninety per cent. of the people do not take care of their teeth. I do hope that this question will have some practical application, but it seems to me that there are many difficulties. It must certainly require a man with a fundamental knowledge of medicine to be of service to a hospital, and I do hope that none but thoroughly competent men may receive hospital appointments.

*Dr. J. Adams Bishop.*—I have brought with me some appliances illustrating a field to which the dentist is especially adapted, in connection with hospital work. These are all splints which have actually done service in the mouth in cases of fractured maxillæ. These cases all represent charity work. I remember one case that appeared to me about seven o'clock in the evening saying that he had broken his lower jaw and wishing to know what could be done. I gave up my night's rest and placed the splint in position next morning. He wore it with comfort for six weeks, when he had it removed, the fracture having healed. I believe this is the most rational and successful way of treating these fractures, and that the majority of such cases can be treated in this way. The first case of this kind was presented at the New York Academy of Medicine in 1863, by Dr. A. L. Sands, who read a paper on the subject. The paper was very favorably discussed. That was in 1863, and since that time I have seen no reason to change my method of procedure in these cases.

We are of great use to the medical profession, as our good friend Dr. Dawbarn knows. There are cases of disfigurement which, after going through our hands, go into their every-day life with the contour of their faces so restored that very few would know what had happened. I think if we would mingle a little more and learn to know each other a little more it would be mutually beneficial.

Dr. Dawbarn suggested one thing that is very useful in a hospital, and that is a tooth-brush. Now, I have a method which is very simple and which I have recommended to a number of the hospital nurses as a substitute for a tooth-brush. It is this thin paper which I have here, by means of which the teeth can be wiped off very successfully.

*Dr. E. Darwin Reed.*—This work is much harder for us than for the physicians. I have been connected with dispensary work since 1894, and am now in the Polheraus Memorial Clinic, Long Island City Hospital, where there are four other dentists. We have had a struggle to get this branch where it belongs. In the first place we find it difficult to get the physicians to turn over cases which belong in our department. Then one's patients do not get the same care in the hospital we would give them. Besides, it is more difficult for us to get the patients to let us do anything for them other than to remove the immediate cause of pain. I have asked some of them to come to my private office to have their teeth cleaned (free); they did not take interest enough in the matter to come. It seems as if we have to educate the physicians as well as the patients in this branch before we can hope for any degree of success.

*Dr. C. A. Brackett.*—The subject has interested me very much. The dental profession has been criticised for not doing more charitable work. It has been said, and perhaps truly, that without considerable rendering of gratuitous service to those in need we are not worthy to be accounted members of a liberal profession. But the practice of dentistry is different from the practice of most other specialties, and it is particularly different from general practice. For the sake of the comparison of the dentist's work with that of the physician or general surgeon I have in mind something like this. Take, for instance, the ordinary ward in a hospital. Say there are twenty-five patients more or less in one large room to be visited by the general practitioner. There can be no combination of circumstances more favorable for the visiting physician than this arrangement. The patients are there together. The attendants and nurses have recorded at brief intervals the developments of the cases, so that it is necessary for the visiting physician to spend only a small amount of time with each patient, glancing over the records and giving to the nurses directions which they are to carry out in the continuance of the treatment. In this way he is enabled to attend to the twenty-five without consuming a great deal of time. To make fit comparison with this the dentist should simply see patients in connection with examination charts which had been prepared previously by others, and his own work in the case would be ended when he had directed others what operations they should perform. The general surgeon comes into the amphitheatre to his patient, who has been prepared by others in every particular. The patient's clothing is arranged, sterilization and etherization accomplished, and he is placed upon the table. All the instruments and appliances are laid out, and as they are successively needed trained assistants put them in the operator's hands. The surgeon performs an operation of very considerable extent and moment,—one of the many marvellous things which have become every-day affairs in recent times,—and the instant the last stitch has been put into the wound he is ready to go, and in most instances that I have seen he does go. The nurses and attendants do the rest. Of course he has examined the case beforehand, made his diagnosis and planned the operation, and he supervises the case afterwards. A similar statement would be more or less true of the specialties generally. But suppose a dental clinic established in the ward of a hospital. The first patient that comes in in the morning has in his mouth the need for attention in the way of dental caries, exposed pulps, alveolar abscess, and pyorrhœa alveolaris sufficient to require the dentist's attention for an aggregate of several solid days of time; and there is no other way in which this service can be rendered than by the dentist's personal labor, if he is to render service equally efficient in his department with that which the physician and surgeon with their many trained assistants accomplish for their patients. It seems to me that this is the situation. It is not much of an exaggeration to say that a single tooth in a condition of such disease as is not rare may require very much more time, effort, and skill on the part of the dentist than a capital operation requires of the surgeon.

The general surgeon who operates in a case of appendicitis needs to be skilled in his special line. He must know the anatomy and the pathology of the parts to be operated upon; but for such an operation, accomplished usually in less than an hour, when he does not do it gratuitously, he gets a fee for which the dentist will work many days, not to say weeks. It is perfectly right that the surgeon should get the higher compensation. The responsibilities are greater, the consequences of incapacity or mistake are vastly more, and there are other thoroughly good reasons why the surgeon should be paid as he is. Doubtless the relation of surgeon's fees and dentist's fees to each other is about as it should be. The point I seek to make is that the surgeon, on account of his larger fees, on account of acquiring experience and gaining and maintaining a reputation, and on account of the prestige of hospital connection, is better able to give considerable portions of his time to gratuitous service than is the dentist. There is a difference. Now, regarding the doing of dental work gratuitously. It seems to me the only practical way of doing such work in a way to adequately meet the needs is in connection with the clinics of our dental schools where there are a large number of learners to do the work. Under these arrangements it is practically the case that each party gets the best of the bargain. Generally speaking, the quality of service is vastly superior to what this class of patients would get from private offices to which they would go. The students are themselves benefited by operating under the supervision and practical teaching of the capable instructors; and so when they graduate and begin private practice the experience which they gained through this system of gratuitous service to those in need becomes one of their best qualifications. Now, I earnestly feel the desirability of the dentist doing his share of charitable work, but, as I have said, ciicumstances are more favorable for physicians and surgeons in this kind of work than they are for dentists. However, it cannot be said that dentists do not do charitable work. They do much charitable work. Every conscientious dentist has patients whom he serves gratuitously. And, again, any one who has anything to do with the relieving of human suffering does a great deal of involuntary gratuitous work, work not intended at the time of its doing to be gratuitous.

If dental clinics are to be established as departments of hospital work, the question arises, To what extent can the service be carried? A visiting dentist on a hospital staff would of course be expected to treat cases of actual suffering in connection with the teeth, to make and apply interdental splints in cases of broken jaw, and generally to perform the minor surgery of the region within which pathological conditions of dental association arise. In a hospital of small size service to this extent would not need to be regular, and it probably would not be heavily burdensome to a dentist who had some leisure; but the maintenance of a regular out-patient dental department affording to all poor and worthy applicants who came full care of the mouths would be a large undertaking.

In Newport we have an excellent hospital. One of the Board of Trustees is here to-night. I hope that we shall hear from him later. The hospital trustees are very desirous of making the institution as largely useful to the community as they can. For years they have maintained a special clinic for the eye and one for the ear, each in charge of a skilled specialist. The trustees feel that if they could establish and maintain a dental clinic suffering humanity would be very much helped. The great difficulty would be to secure competent dentists to do the work. Throughout the year there are in Newport twenty-two thousand inhabitants and sixteen dental practitioners. In the summer there are some thousands more people and four more dentists,—twenty dentists in all. Almost or quite without exception these dentists have full practices. They are men who are very busy, and most of them fill various positions of trust and responsibility, so that they are giving a good deal of time to the service of the community through other channels than their professional work and those dictated by immediate selfishness. It is hard for a dentist who cannot meet in his office all the demands which are made upon him by intelligent, appreciative, well-to-do people to turn such away in order that he may gratuitously serve those less well able to understand that the service is needed. To do for those of the population of the city who might demand gratuitous service all that dentistry can do for those in need might fully occupy all of the time of all the dentists of the place. However, although this is a serious problem, it cannot be said that its solution is impossible or improbable in the near future. Far greater difficulties than this presents have been overcome in numberless instances.

I think that it is the idea of the managers of the Newport Hospital at the outset to make the dental clinic when established a channel of advice and instruction concerning what ought to be done in the care of the teeth both personally and by the dentist, rather than to undertake to give at the clinic all the service which might be needed for full conservation of the organs.

Dentists should be appreciative of the kindly and brotherly spirit with which they are met by the practitioners of general medicine and surgery, and they are appreciative. Certainly in the present state of our specialty, with our limited and incomplete education, it is generous of them to recognize our special attainments in our special work, and it is an honor to be associated with them on anything like equal terms in a public institution for the relief of suffering.

*Dr. G. L. Parmelee.*—It has been a great pleasure to be here to-night. I came not to talk, but to listen. My experience with hospitals has been very limited and mostly in connection with the making of splints for fractures. It seems to me that the time is not ripe for this work in hospitals, as there is not sufficient encouragement, but I can see how we could be of great value to them, by simply relieving suffering, if in no other way.

*Dr. D. A. Fuller.*—I was connected with the Long Island Medical College Hospital, and I think the thing that impressed me most was the way in which the young medical students were glad to come in to my clinic and get information regarding my department. Of course, many of them would practise in the country where this practical knowledge of dentistry would be of great service to them.

*Dr. C. D. Cook.*—I would simply like to call attention to the fact that since the organization of the dental college in Baltimore, the first, I believe, in the world, dentists have used every endeavor to separate their specialty from the general science of medicine as far as possible. This has been the case until comparatively recent times. Now, in England the most far-seeing men of the profession opposed this idea of independent dental colleges with the result that there was established the London Dental Hospital. In this hospital students are educated with the medical students, along the lines of anatomy, physiology, surgery, pathology, and the various branches of medicine. They are students together, they come up for examinations together, and receive their L.D.S. along with the medical students, and for this reason they come more naturally to be associated with the medical men in hospital work. In this country we have nothing like it until later times. The dental education has been, until comparatively recent years, entirely apart from medicine. The dentist has been taught in this country, as we all know, that he is the broadest and best of men, and that what he does not know is not worth knowing. As mechanical men they are very clever, but as scientific men they simply do not compare favorably with medical practitioners, and I think we have ourselves to thank for this, and if our relations with the medical profession is not what we would desire, it is because we have not used our best efforts in that direction.

*Dr. Leo Green.*—It has been my experience in connection with hospital work that the medical staffs are very willing to accord us recognition, but there is always an obstacle in the lack of funds, so that it is necessary not to do missionary work among the physicians, but among the wealthy men who endow hospitals. I think when the value of this work to the poor is appreciated this difficulty may also be overcome.

*Dr. Flanagan.*—I am reminded here of the story of the mate and the captain. It was the mate's duty to keep the log, but as he was frequently intoxicated for days at a time, it fell upon the captain to perform this duty occasionally. Upon recovering from one of his sprees, he found, upon examining the log, this entry made by the captain: "The mate is drunk to-day." He immediately remonstrated with the captain, whereupon the captain said, "Well, it's true, isn't it?" "Ye-es," replied the mate. "Well, we will let it stand," said the captain. The mate went away with a thoughtful air. A few days later, as the captain was examining the log, he discovered this entry. "The captain is sober to-day." Upon questioning the mate, he was met with, "Well, it's true, isn't it?" "Yes, I suppose it is," replied the captain. "Well, let it stand then," said the mate. So I feel like letting everything stand right where it is.

I believe one gentleman spoke of preliminary education. The question of education is so broad that it will never be settled in this world. There is no one system of education that has reached perfection. I do not believe that dentists are more ignorant than physicians. We are all ignorant, in a strict sense.

Regarding the question of acids. We are all groping in the dark regarding this question. There is no doubt that lactic acid does have some effect upon the teeth. The fundamental principle we have not yet discovered.

I would like to ask one question right here: Is it the degree that makes the practitioner, or is it the knowledge? I claim that it is the man and not the degree. There is no specialty or no one system of education that has a mortgage on all the knowledge of the world, because if they had they would foreclose it within twenty-four hours and keep it for themselves. The degree certainly is of no value unless there is an underlying education to bring about results. It has been said that dentists cannot help in hospital work unless they have a thorough knowledge of medicine. I dispute that most emphatically. The world is coming to understand that no man can do many things with his fingers until he has formed a picture in his brain. The arm that moves the world to-day in surgery is not that of the man who works with his brain alone. He is the man who forms the picture with his brain and then does it with his fingers. The man to-day who does the world's best work in dentistry is not the man who tells you how to do something. He is the man who is able to originate and also to perform. It is just the same in dentistry as in surgery. There are many questions coming within the scope of the dental practitioner where his advice would be of great practical value to the physicians, as the treatment of antral troubles, dental abscesses, necrosis, etc., and this advice would be especially valuable in connection with hospital work.

Adjourned.

Fred. L. Bogue, M.D., D.D.S.,
*Editor The New York Institute of Stomatology.*

